# Maternal caregiving burdens and child well-being in an urban informal settlement: an intersectional study of marginalized caste mothers in Chennai, India

**DOI:** 10.3389/fped.2026.1793788

**Published:** 2026-03-31

**Authors:** M. Ananthi, S. Prabakar

**Affiliations:** Department of Social Sciences, School of Social Sciences and Languages, Vellore Institute of Technology, Vellore, Tamil Nadu, India

**Keywords:** child developmental outcomes, India, intersectionality, marginalized communities, maternal caregiving burden, qualitative research, social exclusion, urban informal settlements

## Abstract

**Background:**

Parenting in marginalized urban communities is shaped by intersecting structural inequalities. Limited research examines how caste-based marginalization, gender norms, and poverty collectively influence maternal caregiving practices and child developmental outcomes in urban informal settlement in India. This study investigates the parenting challenges faced by Adi- Dravidar (Scheduled Caste) mothers in Puliyanthope, Chennai, and their implications for child well-being.

**Methods:**

A qualitative study was conducted using semi-structured in-depth interviews with 20 Adi- Dravidar (Scheduled Caste) mothers with children aged 6–11 (Middle Childhood) years residing in Puliyanthope urban informal settlement in Chennai, India. Field observations complemented interview data. Thematic analysis was employed using an intersectional framework to analyzed maternal experiences, coping strategies, and structural constraints affecting parenting practices.

**Findings:**

Findings revealed that all 20 mothers solely bore childcare responsibilities while simultaneously engaging in informal labour, earning ₹8,000–12,000 monthly. Nineteen mothers had minimal formal education below 10th grade. Fathers' involvement in parenting was largely absent, and 90% of households lacked extended family support, leaving mothers isolated in their caregiving roles. Mothers consistently reported chronic time poverty, physical exhaustion, and financial insecurity arising from managing dual responsibilities of paid work and childcare. Limited financial autonomy, unsafe housing conditions, and inability to support children's educational needs further compounded their caregiving burden. Physical punishment was normalized across all families as a perceived protective response to unsafe neighbourhood conditions. Notably, one mother with higher educational attainment demonstrated significantly better capacity to support her children's holistic development, highlighting the protective role of maternal education.

**Conclusion:**

Maternal caregiving in marginalized urban informal settlement contexts is profoundly constrained by intersecting structural inequalities rooted in caste, gender, and poverty. These constraints not only impose severe physical, emotional, and economic burden on mothers themselves but also directly impact child developmental outcomes, educational attainment, and overall well-being. Findings underscore the urgent need for multi-level interventions, including community-based childcare services, economic empowerment and mental health support programs for mothers, promotion of shared parenting responsibilities, and gender-sensitive policies that address both maternal caregiving burden and the intergenerational transmission of disadvantage in urban informal settlements in India contexts.

## 1 Introduction

Parenting practices fundamentally shape children's emotional, cognitive, and social development (1). Globally, mothers residing in urban informal settlements and marginalized communities face intersecting challenges of poverty, social exclusion, and inadequate institutional support that profoundly constrain their caregiving capacities and children's developmental outcomes (2). Research across low- and middle-income countries consistently documents how structural inequalities rooted in gender, ethnicity, and socioeconomic status compound maternal caregiving burdens in marginalized urban informal settlement contexts (3). However, scholarly engagement with parenting remains uneven, with substantially less attention devoted to understanding how impoverished and marginalized mothers raise children in unsafe, high-stress environments compared to families in stable households (4). This imbalance reflects implicit assumptions within existing literature that obscure the lived realities of caregiving in structurally deprived contexts, notably, that parenting practices are shaped primarily by individual parental behaviour and choice rather than by structural forces such as poverty, caste discrimination, and gender inequality, where mothers negotiate child-rearing amid persistent discrimination, material insecurity, and limited institutional support.

Globally, an estimated 1.1 billion people reside in urban informal settlements, with India accounting for approximately 65 million urban informal settlement dwellers (5). Scheduled Caste communities constitute approximately 16.6% of India's total population according to census data (6) and continue to face compounded disadvantages rooted in historical caste discrimination that shapes access to education, employment, and social mobility (7). Children residing in urban informal settlements face disproportionate risks across multiple dimensions, including inadequate access to clean water, sanitation, and adequate nutrition, with rates of deprivation significantly higher in marginalized urban communities (8).

In India, parenting research has largely concentrated on urban middle-class families, leaving the experiences of mothers in marginalized urban communities significantly understudied (9–11). Although studies on Adi-Dravidar (Scheduled Caste) have extensively documented patterns of social exclusion and discrimination, the everyday parenting experiences of Adi-Dravidar (Scheduled Caste) mothers residing in stigmatized urban neighbourhoods remain largely absent from scholarly analysis (12–14).

India's caste system represents a hierarchical social structure that has historically relegated certain communities to the lowest social strata, subjecting them to severe discrimination, social exclusion, and economic deprivation. Dalits, officially designated as Scheduled Castes under Indian Constitutional law, constitute these historically marginalized communities. In Tamil Nadu, Scheduled Caste communities are specifically referred to as Adi-Dravidar (Scheduled Caste), a term used in state government policy and administration. The Adi-Dravidar (Scheduled Caste) community in Puliyanthope, Chennai, continues to experience intersecting forms of caste-based discrimination, urban poverty, and social stigma that profoundly shape their everyday lives, including parenting experiences (7).

Urban informal settlements in India are characterised by inadequate housing, limited access to potable water, poor sanitation, and chronic food insecurity, conditions placing residents, particularly children, at heightened risk of illness and malnutrition (15–17). Beyond physical constraints, urban informal settlement is socially constructed as spaces inhabited by “undeserving” populations, frequently associated with criminality and moral failure, thereby subjecting residents to persistent social stigma (18). In settlements such as Puliyanthope, this stigma is reinforced through ongoing infrastructural neglect and unsafe living environments (19). Research on parenting practices in urban informal settlement communities consistently indicates that mothers bear primary responsibility for childcare and household management (20, 21). Time constraints and economic pressures significantly limit parental involvement with children, while fathers' engagement in day-to-day caregiving remains minimal (22, 23). Mothers frequently combine domestic responsibilities with informal labour, resulting in physical exhaustion and limited capacity for sustained child-centred interaction (24, 25). Traditional gender norms continue to shape parenting dynamics, with fathers conceptualizing breadwinning as their sole parental responsibility (26, 27).

Parenting research has historically examined child-rearing as a shared parental responsibility. However, in contexts of intersecting caste-based marginalization, gender inequality, and poverty, this assumption does not reflect lived reality. While parenting involves both mothers and fathers, research consistently demonstrates that in marginalized communities, mothers bear disproportionate responsibility for childcare, household management, and children's emotional well-being (3). This gendered division of caregiving is further intensified by structural inequalities such as poverty and caste discrimination, which limit fathers' involvement while simultaneously increasing mothers' caregiving burden. In urban informal settlements specifically, mothers routinely combine physically demanding paid labour with the full burden of unpaid caregiving, with minimal or no paternal contribution, making maternal caregiving a distinct and critical unit of analysis rather than simply one dimension of broader parenting research. Focusing specifically on maternal caregiving, therefore, provides a critical lens through which to examine how intersecting structural inequalities shape children's developmental outcomes in marginalized urban communities (28).

Despite this growing body of literature, relatively few studies focus specifically on the Adi-Dravidar (Scheduled Caste) population or systematically examine how caste, labour, and urban informal settlement marginalization intersect to shape parenting experiences. The parenting experiences of Adi-Dravidar (Scheduled Caste) mothers in Puliyanthope, one of Chennai's most marginalized and socially stigmatized communities, remain largely absent from empirical scholarship. This omission significantly limits understanding of how structural inequalities shape parenting practices within urban informal settlements in Adi-Dravidar (Scheduled Caste) contexts and their implications for child well-being.

### 1.1 Research questions

This study examines maternal caregiving experiences and their impact on child well-being among Adi- Dravidar (Scheduled Caste) families in Puliyanthope urban informal settlement, Chennai. The study was guided by the following research questions:

1. What structural challenges do mothers from Adi-Dravidar (Scheduled Caste) communities experience in fulfilling parenting responsibilities and supporting child development?
2. How do mothers in marginalized urban informal settlement communities manage the dual burden of paid work and childcare responsibilities?

### 1.2 Theoretical framework

This study employs an intersectional framework, which examines how multiple and overlapping forms of inequality shape lived experiences (29). Intersectionality emphasizes that caste, gender, and poverty do not operate as separate factors but intersect to produce compounded disadvantages in everyday life. In the Indian context, Dalit women experience layered marginalization as members of historically stigmatized caste groups, as women within patriarchal social structures, and as residents of economically deprived urban environments (30). These intersecting forms of inequality influence access to education, employment, social support, and institutional resources while simultaneously intensifying women's unpaid caregiving responsibilities.

An intersectional framework is particularly relevant for understanding motherhood in urban informal settlement, where parenting practices are shaped by structural constraints, social exclusion, and economic insecurity. By adopting this perspective, the present study situates mothers' parenting experiences within broader systems of caste-based discrimination, gendered norms, and poverty, rather than attributing caregiving challenges to individual limitations.

Intersectionality is particularly applicable to the present study because Adi-Dravidar (Scheduled Caste) mothers in Puliyanthope occupy multiple marginalized positions simultaneously, as Dalit women, as residents of a stigmatized urban informal settlement, and as informal sector workers living in poverty. No single axis of analysis, whether gender, caste, or class alone, can adequately capture the complexity of their caregiving experiences.

In practice, this framework guided the analysis by prompting examination of how caste discrimination, gendered caregiving norms, and economic precarity intersect to shape mothers' daily parenting practices, their access to support systems, and ultimately children's developmental outcomes in Puliyanthope.

## 2 Materials and methods

### 2.1 Study design

This study adopted a qualitative research design. Specifically, semi-structured in-depth interviews complemented by field observations were employed to examine maternal caregiving practices and child well-being in a marginalized urban informal settlement. The study is guided by an intersectional framework, which examines how caste, gender, and poverty simultaneously shape maternal caregiving experiences. Intersectionality was employed as both a theoretical lens and an analytical tool to examine how these overlapping structural inequalities shape the lived experiences of Adi-Dravidar (Scheduled Caste) mothers in Puliyanthope (29).

### 2.2 Study setting

The research was conducted in Puliyanthope, a socioeconomically disadvantaged neighbourhood in North Chennai, Tamil Nadu, India. Puliyanthope is characterized by inadequate infrastructure, insecure livelihoods, substandard housing (predominantly thatched roofs and asbestos-sheeted structures), poor sanitation, and a long history of caste-based marginalization. The settlement was purposively selected due to its sustained institutional neglect and concentration of Adi-Dravidar (Scheduled Caste) residents, rendering it a critical context for analyzing parenting practices within marginalized urban environments. The target population included working mothers from the Adi Dravidar (Scheduled caste) community with at least one child aged 6–11 years.

### 2.3 Participants and sampling

The study population consisted of working mothers from the Adi-Dravidar (Scheduled Caste) Hindu community with at least one child aged 6–11 years (Middle-Childhood). Purposive sampling (31) was employed to identify 20 participants meeting the inclusion criteria. This non-probability sampling approach was appropriate for qualitative inquiry, enabling the selection of participants with relevant lived experiences and facilitating the generation of rich, context-specific insights. Data saturation (32) was reached at interview 16, with interviews 17 through 20 confirming saturation as no new information emerged. This is also reflected in the findings section of the manuscript. No participants withdrew or dropped out; all 20 completed the full interview.

Access to participants was facilitated through a local NGO with an established presence in Puliyanthope, which served as the community gatekeeper. The researcher communicated the study's inclusion criteria to the NGO before data collection, and the NGO identified and suggested eligible participants accordingly. This approach ensured both the safety of the researcher and the willingness and comfort of participants before any interview commenced.

### 2.4 Inclusion criteria

- Mothers belonging to the Adi- Dravidar (Scheduled Caste) Hindu community
- Mothers with at least one child aged 6–11 (Middle Childhood) years
- Mothers currently engaged in any form of paid labour

### 2.5 Exclusion criteria

- Single mothers
- Mothers with children below 6 years of age and after 11 years

Children aged 6–11 years, corresponding to the middle childhood developmental stage, were specifically selected as this period represents a critical phase of cognitive, social, and emotional development. During middle childhood, parental involvement, particularly maternal engagement, plays a pivotal role in shaping children's educational outcomes, social competencies, and overall well-being. Furthermore, this age group coincides with formal schooling, making maternal caregiving support particularly consequential for children's developmental trajectories.

### 2.6 Data collection

Data were collected between 5 and 14 March 2025 through semi-structured in-depth interviews and field observations. Data saturation (32) was reached with the 16th interview, with no new themes identified, indicating sufficient depth and breadth of data collection. An interview guide containing over 20 open-ended questions explored participants' daily routines, caregiving practices, physical and emotional stress, access to support networks, and overall well-being. Interviews were conducted in Tamil, the participants' native language, to facilitate open and comfortable communication.

Given participants' demanding work schedules, interviews were primarily conducted during late evening hours following their return from work. While initial engagement proved challenging due to physical exhaustion, sustained presence in the community and informal interactions facilitated rapport-building and trust, ultimately enabling meaningful data collection. Researchers remained sensitive to participants' emotional well-being throughout interviews, particularly during discussions of sensitive topics, ensuring a supportive and respectful environment at all times.

Field observations documented participants' everyday lives, including work patterns, domestic environments, and mother-child interactions. Observational data captured non-verbal cues, living conditions, and behavioural patterns not always explicitly articulated during interviews, thereby enriching the qualitative dataset. Following initial data collection, the researcher returned to the community to conduct observational verification, ensuring emerging interpretations were grounded in participants' lived realities.

### 2.7 Data analysis

Data were analyzed using thematic analysis following Braun and Clarke's (33) six-phase framework. An inductive coding approach identified recurring patterns and themes related to caregiving practices, coping strategies, emotional labour, and structural constraints. Field notes from observational data were triangulated with interview transcripts to enhance analytical depth and strengthen the validity of findings.

The analytical process involved: (1) familiarization with data through repeated reading of transcripts, (2) generation of initial codes, (3) searching for themes, (4) reviewing themes, (5) defining and naming themes, and (6) producing the analytical report. This systematic approach ensured findings accurately reflected the lived realities of parenting within Adi-Dravidar (Scheduled Caste) households.

Coding of transcripts was conducted by the lead researcher using an inductive approach. To ensure analytical rigour, preliminary codes and identified themes were regularly discussed with and reviewed by the research supervisor, with any interpretive discrepancies resolved through ongoing dialogue and consensus.

### 2.8 Translation and verification

All participants provided consent for audio recording. All interviews were transcribed verbatim in Tamil by the lead researcher, who possesses native fluency in Tamil and professional proficiency in English. Transcripts were subsequently translated into English by the lead researcher to ensure linguistic accuracy and contextual fidelity. To ensure translation reliability, transcripts were re-read multiple times and reviewed by the research supervisor, who verified the accuracy of translated transcripts against original Tamil recordings.

### 2.9 Ethical considerations

Ethical approval was obtained from the Institutional Ethics Committee for Human Studies, Vellore Institute of Technology (Approval No: VIT/IECHH/2024/15 IECH/15 June 2024/27, dated 13 July 2024, valid until 12 July 2025) and Don Bosco Service Society (NGO).

Given varying literacy levels among participants, verbal informed consent was secured from all mothers prior to data collection. Research objectives, procedures, and participants' rights were explained in Tamil, their native language. Confidentiality and anonymity were rigorously maintained through the use of numerical code (participant 1–20), throughout the manuscript. Audio recordings and transcripts were stored securely on a password-protected device, accessible only to the lead researcher and research supervisor.

Participation was entirely voluntary, and participants were informed of their right to withdraw at any stage without adverse consequences. The research team maintained sensitivity to participants' demanding schedules and emotional well-being throughout data collection. Given that participants navigated poverty, caste-based discrimination, and gender inequality as part of their everyday lived experience, the research team remained attentive to both visible and non-visible signs of distress throughout data collection. While participants engaged openly and willingly, the research team acknowledges that emotional distress may not always be outwardly expressed during interviews and may emerge after discussions of sensitive topics conclude. Participants were reminded of their right to withdraw at any point, and the researcher maintained a supportive presence throughout to minimize potential harm.

### 2.10 Researcher reflexivity

The research team acknowledges positionality differences between researchers and participants. As educated, English-speaking researchers from higher socioeconomic backgrounds, we recognize that these power differentials may have influenced participants' responses. Interviews were conducted in Tamil to minimize power imbalances, and a non-judgmental approach was maintained throughout. In accordance with institutional guidelines, financial compensation was not provided to participants. We recognize this as a significant ethical tension, particularly given participants' severe financial constraints. The absence of compensation, combined with interviews conducted during late evening hours when participants were physically exhausted after full working days, raises important questions about the nature of voluntary participation and potential invisible power dynamics. Participants may have felt a sense of obligation to engage despite fatigue and financial vulnerability. We acknowledge that these conditions may have influenced the depth and nature of responses. To mitigate these dynamics, the researcher maintained a flexible and participant-led interview approach, prioritized rapport-building over data extraction, and consistently reminded participants of their right to withdraw without consequence.

The lead researcher's Master's background in Gender Studies involved two significant fieldwork experiences that directly informed this study. First, the researcher alongside classmates organized and delivered awareness programmes for marginalized community members in Dhayanoor village, Tiruchy. Second, through the Rashtriya Uchchatar Shiksha Abhiyan (RUSA) project, the researcher conducted data collection across Ariyalur and Perambalur districts. Both experiences cultivated deep sensitivity to caste-based discrimination, poverty, and gender inequality within marginalized communities, directly informing the research design, community engagement approach, and analytical framework of the present study.

## 3 Results

### 3.1 Sociodemographic characteristics

Twenty mothers from the Adi-Dravidar (Scheduled Caste) Hindu community participated in the study. Sociodemographic characteristics are presented in Table 1.

**Table 1:** Sociodemographic characteristics of participants (*N* = 20).

Characteristic	*n*	%
Maternal Age		
32–45 years	8	40.0
Not reported	12	60.0
Number of Children		
2 children	17	85.0
3 or more children	3	15.0
Gender of Children (Aged 6–11 years)		
Boys and girls (mixed)	20	100.0
Maternal Education		
No formal education	10	50.0
Primary (1st–5th grade)	3	15.0
Middle school (6th–9th grade)	6	30.0
Bachelor’s degree (Engineering)	1	5.0
Paternal Education		
No formal education	11	55.0
Primary–middle school	6	30.0
Secondary (10th grade)	2	10.0
IT diploma	1	5.0
Maternal Occupation		
Domestic/housekeeping worker	14	70.0
Sanitation worker	5	25.0
Engineering professional	1	5.0
Housing Type		
Thatched roof	7	35.0
Asbestos sheets	10	50.0
Concrete/semi-permanent	3	15.0
Family Structure		
Nuclear (without extended family)	18	90.0
Extended (with grandparents)	2	10.0
Household Size		
4–6 members	20	100.0
Monthly Household Income (INR)		
Below ₹20,000	12	60.0
₹20,001–₹30,000	6	30.0
Above ₹30,000	2	10.0
Maternal Monthly Income (INR)		
₹8,000–₹12,000	19	95.0
Above ₹12,000	1	5.0

Age data available for 8 participants only; 12 participants were unable to recall their exact age.

Most women (95%) had not completed secondary schooling. Only one participant held a Bachelor of Engineering degree. Fathers' educational attainment was similarly low, with 55% having no formal education. The majority of households (90%) followed nuclear family structures without grandparent support. Most families (85%) lived in thatched or asbestos-sheeted housing, reflecting low socioeconomic status and material vulnerability. Combined household income remained below ₹30,000 monthly for 95% of families.

### 3.2 Findings

Six major themes were identified from data analysis: (1) gendered division of caregiving labour, (2) economic insecurity and financial autonomy, (3) educational engagement and aspirations, (4) Physical Punishment as a Normalized Parenting Practice in Unsafe Living Environments, (5) social isolation and absent support systems, and (6) maternal resilience amid structural constraints.

#### 3.2.1 Theme 1: gendered division of caregiving labour

##### 3.2.1.1 Exclusive maternal responsibility for childcare

Across all 20 households, mothers assumed sole responsibility for childcare, household management, and children's education despite engaging in paid labour outside the home. Fathers were largely disengaged from children's everyday lives, typically leaving for work and returning home primarily to rest.

“I wake at 4 AM to prepare meals, get children ready for school, then work until evening. When I return, I must cook again, help with homework, and manage everything. My husband rests after work. He says earning money is his responsibility, children are mine.” (Participant 8, housekeeping worker, mother of two)

Participant 8's account powerfully illustrates how maternal caregiving in Puliyanthope is not a matter of individual choice, but a structural reality shaped by the intersection of gender, caste, and poverty. Her 4 AM start, preparing meals, managing school routines, working a full day, then returning to cook, clean, and supervise homework, reflects a daily cycle of exhaustion that leaves no space for rest or self-care. Mothers like Participant 8 are simultaneously required to participate in paid labour and absorb the full burden of unpaid caregiving, with no expectation of paternal contribution. This reflects deeply entrenched patriarchal norms reinforced by caste-based economic marginalization that leave Adi-Dravidar (Scheduled Caste) mothers with no alternative but to manage both spheres entirely alone.

##### 3.2.1.2 Absent paternal engagement

Only one or two fathers reportedly engaged with children, and such engagement was limited to shared mealtimes. Children predominantly turned to mothers to express emotional concerns, even when mothers had limited time or physical energy. This pattern reflects deeply entrenched patriarchal norms positioning men as breadwinners and women as caregivers, a dynamic observed across urban informal settlements in Tamil Nadu.

“My husband goes to work in the morning and returns in the evening. He eats and sleeps. But when I return from work, I must still cook, clean, and care for the children. He never helps.” (Participant 2, housekeeping worker, mother of two)

Participant 2's account reflects a pattern observed consistently across all 20 households. Fathers' disengagement from domestic and caregiving responsibilities is not simply individual neglect but a product of patriarchal norms that define masculinity through breadwinning alone. For Adi-Dravidar (Scheduled Caste) mothers, this burden is further intensified by caste-based discrimination that confines them to low-status, physically demanding occupations, leaving them exhausted yet solely responsible for all caregiving demands upon returning home.

##### 3.2.1.3 Time poverty and physical exhaustion

Mothers experienced chronic time poverty, physical exhaustion, and emotional strain. The dual burden of paid and unpaid labour left minimal capacity for sustained child-centred interaction or self-care.

“After working all day cleaning houses, my body aches. But I must still care for my children, cook, clean our home. There is no rest for mothers like us.” (Participant 14, sanitation worker, mother of two)

Participant 14's account captures the chronic physical exhaustion experienced by mothers navigating the dual burden of paid and unpaid labour. This time, poverty is not simply a personal hardship, but a structural condition produced by the intersection of gender inequality, caste-based occupational segregation, and poverty. With no paternal support, no extended family assistance, and no institutional childcare, mothers absorb all caregiving responsibilities regardless of their physical and emotional capacity, severely limiting meaningful child-centred interaction.

#### 3.2.2 Theme 2: poverty and male financial irresponsibility compounding maternal caregiving burden

##### 3.2.2.1 Precarious income and irregular employment

Nineteen mothers (95%) worked as daily wage labourers in domestic work or sanitation, earning ₹8,000–12,000 monthly. Income was irregular and insufficient for basic household needs, leaving no scope for savings or long-term financial planning.

“Some days there is work, some days not. How can we save when we struggle to afford daily food?” (Participant 11, housekeeping worker, mother of two)

Participant 11's account reflects the precarious economic reality faced by the majority of mothers in this study. As daily wage labourers confined to low-status occupations through caste-based occupational segregation, these women have no financial security or ability to plan for their children's futures. The intersection of caste discrimination, gender inequality, and poverty creates a cycle of economic vulnerability that is structurally imposed rather than individually chosen, with direct consequences for children's nutrition, education, and overall well-being.

##### 3.2.2.2 Limited financial autonomy

Although government initiatives facilitated opening bank accounts for educational purposes, most mothers lacked meaningful financial autonomy. In many cases, husbands controlled bank accounts. Only one mother (The Engineering Graduate) reported independently managing finances and actively saving for her child's education.

“I do not have my ATM card with me. My husband handles all the money. I have no access to our account.” (Participant 4, housekeeping worker, mother of two)

Participant 4's account illustrates how financial exclusion operates as a form of domestic control rooted in patriarchal norms. Despite actively earning income, her inability to access her own earnings reflects the intersection of caste, gender, and poverty that strips Adi-Dravidar (Scheduled Caste) women of economic autonomy. This directly impacts children's well-being, as mothers' capacity to meet children's educational and nutritional needs becomes entirely dependent on male cooperation.

##### 3.2.2.3 Male financial irresponsibility

Economic insecurity was exacerbated by limited male financial contribution. According to NGO workers and participants, extramarital relationships and alcohol use among men were common, leaving women solely responsible for childcare, household expenses, and debt repayment.

“My husband drinks. Sometimes he gives money, sometimes not. I must manage everything, children's food, school fees, rent. The burden is mine alone.” (Participant 13, sanitation worker, mother of two)

Participant 13's account captures the compounded financial burden experienced by Adi-Dravidar (Scheduled Caste) mothers when male financial irresponsibility intersects with caste-based economic marginalization. Her inability to rely on her husband's income, consumed by alcohol use, means she alone must manage food, school fees, and rent on an irregular daily wage. This reflects systemic economic disadvantage rooted in the intersection of caste and gender, where women bear dual burdens of paid and unpaid labour while being constrained from financial autonomy, perpetuating cycles of dependency and vulnerability.

#### 3.2.3 Theme 3: maternal education as a determinant of children's developmental opportunities

##### 3.2.3.1 Maternal responsibility for educational support

Most children (95%) attended government schools, with only the engineering graduate's children enrolled in private schools. Mothers assumed sole responsibility for school-related matters, including monitoring academic progress, attending parent-teacher meetings, and addressing disciplinary concerns. Fathers were largely absent from children's educational lives.

Mothers' determination to educate their children must be understood within the context of generational caste discrimination. Having been denied educational opportunities themselves due to intersecting caste and gender inequalities, mothers recognized education as the primary pathway to breaking cycles of caste-based marginalization. However, mothers consistently emphasized that one parent's effort alone is insufficient; meaningful community empowerment requires active involvement and support from both parents in children's education.

##### 3.2.3.2 Impact of maternal education on child development

The mother with higher education demonstrated markedly different parenting practices, actively supporting her children's holistic development through enrolment in extracurricular activities (Swimming, Boxing, Silambam). Her children earned multiple medals and certificates. Her sustained involvement illustrates how maternal educational attainment generates substantial intergenerational benefits.

“I ensure my children participate in sports and studies. Education changed my life. I want the same for them.” (Participant 6, engineering graduate, mother of two)

Participant 6's experience powerfully demonstrates how maternal educational attainment transforms children's developmental opportunities. Her ability to actively engage in her children's holistic development stands in sharp contrast to the experiences of other mothers in the study. This contrast must be understood within an intersectional lens; the majority of Adi-Dravidar (Scheduled Caste) mothers were denied educational access due to generational caste discrimination and gender inequality, creating compounding disadvantages that are directly reproduced in their children's developmental outcomes.

##### 3.2.3.3 Financial constraints limiting extra-curricular opportunities

In contrast, most mothers reported financial constraints significantly limiting children's participation in extracurricular activities. Initial engagement in sports or arts was frequently discontinued due to costs.

“My daughter wanted to learn dance, but we cannot afford the fees. Government school is free, but other activities require money we don't have.” (Participant 17, housekeeping worker, mother of two)

Participant 17's account illustrates how poverty operates as a direct barrier to children's holistic development. While mothers demonstrated strong aspirations for their children's growth beyond academic education, financial constraints systematically prevented these aspirations from being realized. This must be understood within an intersectional framework; caste-based occupational segregation confines Adi-Dravidar (Scheduled Caste) mothers to low-wage labour, leaving insufficient income for anything beyond basic survival needs, directly limiting children's access to developmental opportunities available to children in more economically stable households.

##### 3.2.3.4 Parental educational limitations

Several mothers with limited education reported difficulties supporting children's learning due to their own lack of foundational skills.

“I cannot read well. When my son asks homework questions, I feel helpless. I want to help but don't know how.” (Participant 10, sanitation worker, mother of two)

#### 3.2.4 Theme 4: physical punishment as a normalized parenting practice in unsafe living environments

##### 3.2.4.1 Normalization of physical punishment

Physical punishment was reported across all 20 households, with both parents acknowledging the use of physical force. Parents justified this practice by stating that children would not comply otherwise. Normalisation of physical punishment was closely linked to anxieties regarding neighbourhood safety.

“We must be strict. Outside is dangerous. If we don't discipline them strongly, they won't stay safe at home.” (Participant 5, sanitation worker, mother of two)

Participant 5's account reveals how physical punishment in this context cannot be reduced to individual parenting failure. Rather, it reflects a survival response to structural violence; the intersection of poverty, unsafe neighbourhoods, and institutional neglect creates an environment where parents perceive strict physical discipline as the only available protective mechanism. For Adi-Dravidar (Scheduled Caste) mothers already navigating caste-based marginalization and gender inequality, fear for children's safety becomes an additional burden layered onto their existing caregiving challenges.

##### 3.2.4.2 Pervasive safety concerns

A few participants described the area as unsafe, referring to child abuse incidents. Fear and insecurity were pervasive. Parents often restricted children's mobility and prohibited outdoor play as protective measures. Media reports of sexual violence against children in the region corroborated these fears. While physical punishment is recognized as harmful to child well-being in child development discourse, in this context, it appeared to stem from deeply internalized vulnerability rather than negligence.

##### 3.2.4.3 Intergenerational transmission of non-cooperative behaviour

For parents living under conditions of instability and constant threat, physical discipline became incorporated as a tool perceived to ensure obedience and safeguard children. NGO practitioners reported that non-cooperative behaviour among children was common and linked to absent cooperative parenting relationships within households. Children replicated fathers' disengagement and resistance toward mothers, displaying non-compliance. This intergenerational transmission reflects how children internalize and reproduce observed power relations.

“Children see fathers ignore mothers' requests. Then children also don't listen to mothers. The pattern repeats.” (NGO worker, 24 years old)

Addressing punitive parenting requires understanding these practices within broader structural violence experienced by communities. When poverty, caste-based marginalization, and institutional neglect converge, harmful disciplinary practices become normalized.

#### 3.2.5 Theme 5: compounded isolation intensifying maternal caregiving burden

##### 3.2.5.1 Limited extended family support

Only two households (10%) included grandparent co-residence, and even in these cases, involvement in childcare was minimal. The majority (90%) followed nuclear family structures without extended family support.

“We have no grandparents nearby to help. It's only me managing children while working. I feel alone in this struggle.” (Participant 19, housekeeping worker, mother of two)

Participant 19's account reflects the profound isolation experienced by the majority of mothers in this study. The absence of extended family support is not simply a domestic inconvenience, but a structural condition intensified by urban migration patterns and poverty that have fragmented traditional community support networks. For Adi-Dravidar (Scheduled Caste) mothers already bearing the dual burden of paid and unpaid labour, the absence of grandparental support leaves them entirely alone in managing caregiving responsibilities, with direct consequences for both maternal well-being and children's developmental outcomes.

##### 3.2.5.2 Maternal isolation and lack of respite

Limited extended family support intensified mothers' caregiving responsibilities, particularly in contexts already shaped by poverty and marginalization. When combined with absent paternal involvement, mothers experienced profound social isolation.

“If I am sick or exhausted, there is no one to help. I must still care for children. Who else will do it?” (Participant 7, housekeeping worker, mother of two)

Participant 7's account captures the profound isolation experienced by Adi-Dravidar (Scheduled Caste) mothers in this study. This isolation cannot be understood through the lens of poverty alone. While financial hardship limits access to paid childcare, the absence of support networks among these mothers is further compounded by caste-based spatial segregation that has historically concentrated Adi-Dravidar communities in stigmatized neighbourhoods with weakened institutional and community support systems (7, 19). Research on Dalit women documents how caste-based social exclusion erodes community solidarity and access to support networks in ways that extend beyond economic deprivation (14). For Adi-Dravidar mothers already navigating the dual burden of paid and unpaid labour, this caste-specific social isolation leaves them entirely alone in managing caregiving responsibilities, with direct consequences for both maternal well-being and children's developmental outcomes.

##### 3.2.5.3 Absence of institutional support

The absence of affordable childcare services compelled many mothers to leave their children unsupervised during work hours or in the care of older siblings. This created safety concerns, as support left mothers managing multiple demands in isolation.

#### 3.2.6 Theme 6: maternal resilience amid structural constraints

##### 3.2.6.1 Unwavering commitment to children's well-being

Despite overwhelming challenges, mothers demonstrated a strong commitment to children's well-being. They prioritized children's school attendance and basic necessities even while experiencing chronic exhaustion and financial strain.

“No matter how tired I am, I ensure my children eat and attend school. Their future depends on education. I will sacrifice anything for them.” (Participant 3, sanitation worker, mother of three)

Participant 3's account demonstrates the remarkable resilience of Adi-Dravidar (Scheduled Caste) mothers who maintain an unwavering commitment to their children's well-being despite overwhelming structural constraints. However, this resilience must not be romanticized; it operates within severe limitations imposed by poverty, caste discrimination, and absent support systems. Framing maternal resilience solely as individual strength risks obscuring the urgent need for structural interventions that address the root causes of marginalization rather than placing the entire burden of children's wellbeing on exhausted mothers.

##### 3.2.6.2 Coping strategies under constraint

Mothers employed various coping strategies, including reducing personal expenses, seeking informal loans, and working multiple jobs to meet children's needs. However, these strategies proved insufficient to overcome structural barriers affecting child development and well-being.

“I work in sanitation and also do housekeeping work in other people's homes. Whatever it takes to manage my children's needs.” (Participant 9, sanitation worker, mother of two)

Participant 9's account illustrates how mothers' coping strategies, while demonstrating extraordinary resilience, remain fundamentally insufficient to overcome structural barriers. Working multiple jobs addresses immediate financial needs but simultaneously reduces the time and energy available for meaningful child-centred interaction. This paradox reflects the impossibility of individual coping strategies substituting for systemic support, without structural interventions addressing caste-based poverty, gender inequality, and institutional neglect, maternal resilience alone cannot adequately protect children's developmental outcomes.

##### 3.2.6.3 Resilience within systemic limitations

The study revealed how maternal resilience operates within severe constraints. While mothers worked tirelessly to protect and nurture children, their efforts were continually undermined by poverty, absent support systems, unsafe neighbourhoods, and institutional neglect. This underscores that individual resilience cannot substitute for systemic support.

## 4 Discussion

This study provides critical insights into maternal caregiving experiences and child well-being outcomes among Adi-Dravidar (Scheduled Caste) families in an urban informal settlement context. Findings reveal how intersecting structural inequalities rooted in caste, gender, and poverty profoundly constrain parenting practices and child development.

### 4.1 Intersecting structural inequalities

The intersection of caste-based marginalization, gendered caregiving expectations, and economic precarity created compounded disadvantages for mothers and children. These findings align with intersectionality theory, which posits that multiple forms of inequality operate simultaneously to produce unique patterns of disadvantage (29, 30). The study demonstrates that maternal and child outcomes cannot be understood by examining gender, caste, or class in isolation. Rather, their intersection creates specific vulnerability patterns requiring targeted interventions. Critically, this study extends intersectionality scholarship by demonstrating that caste operates not merely as a socioeconomic marker but as an independent axis of discrimination that restricts personhood and civic participation in ways that poverty alone cannot explain (7), with direct consequences for maternal caregiving capacity and children's developmental outcomes.

Caste-based discrimination played a significant role in shaping mothers' economic vulnerability. As Adi-Dravidar (Scheduled Caste) women, participants faced double marginalization rooted in both their caste identity and gender. This intersecting discrimination historically denied them access to education and confined their employment opportunities to low-status occupations such as sanitation and domestic housekeeping, occupations traditionally assigned to the Scheduled Caste communities. This caste-based occupational segregation perpetuates cycles of poverty and economic dependency across generations.

Critically, the economic vulnerabilities experienced by these mothers cannot be reduced to poverty alone. Non-Dalit poor mothers in India face significant gender-based hardships; however, Adi-Dravidar (Scheduled Caste) mothers in Puliyanthope face an additional, caste-specific layer of exclusion that operates independently of poverty. As documented in Puliyanthope (19), Adi-Dravidar residents are denied post-paid data connections, rejected for government loans, and refused marriage alliances solely upon disclosure of their area and caste identity. Children from this community are subjected to caste-based slurs at inter-school competitions, and residents report being treated as “untouchables” when disclosing their origins in educational institutions. These forms of exclusion, rooted in caste stigma rather than poverty alone, compound the caregiving burdens of Adi-Dravidar mothers in ways that are structurally distinct from the experiences of non-Dalit poor mothers. While poverty restricts resources, caste discrimination restricts personhood, dignity, and access to basic civic participation, creating a qualitatively different experience of marginalization.

These findings carry important implications for how researchers and policymakers conceptualize disadvantage in urban India. Conventional poverty-reduction frameworks that treat economic deprivation as the singular driver of poor maternal and child outcomes risk misdiagnosing the problem and, consequently, prescribing insufficient solutions. In the Puliyanthope context, even modest improvements in household income are unlikely to fully dismantle the caste-based stigma, occupational restrictions, and civic exclusion that compound maternal caregiving burdens. This study therefore calls for an explicitly intersectional policy architecture, one that simultaneously addresses income support, caste discrimination, and gendered labour norms rather than treating these as separate programmatic domains. Future quantitative studies employing intersectional analytic models across larger, more diverse samples would further strengthen the evidence base for such integrated approaches (7, 30).

### 4.2 Gendered caregiving and absent paternal involvement

In the Adi-Dravidar (Scheduled Caste) households studied, gendered caregiving divisions were further intensified by caste-based occupational segregation that confined mothers to physically exhausting low-wage labour, leaving them with depleted capacity for sustained child-centred interaction despite their unwavering commitment to their children's wellbeing. This pattern reflects deeply entrenched gender norms positioning childcare as women's domain and breadwinning as men's sole contribution (26, 27). Fathers' absence from children's educational and emotional lives has significant implications for child development and maternal well-being. Research demonstrates that paternal involvement positively influences children's cognitive development, academic achievement, and socio-emotional well-being (34, 35). Conversely, absent paternal engagement compounds maternal stress and limits children's access to diverse caregiving relationships. These findings underscore the urgent need for gender-transformative interventions that address the structural roots of gendered caregiving divisions rather than simply encouraging fathers to participate more.

The absence of paternal involvement observed in this study is not merely a cultural preference, but a structurally reproduced outcome shaped by the intersection of caste-prescribed occupational roles and hegemonic masculine norms that equate male identity with economic provision rather than emotional nurturing. This is particularly consequential during middle childhood, a developmental stage in which children require consistent emotional scaffolding, identity formation support, and academic engagement from both caregivers (34). When fathers are structurally excluded from caregiving roles, mothers are left as sole providers of emotional labour on top of paid work, an unsustainable burden that inevitably compromises the depth and quality of maternal engagement. Interventions that focus only on attitudinal change among fathers, without challenging the caste-based labour structures that trap them in extractive, time-intensive employment, are unlikely to achieve meaningful shifts in caregiving equity. A structural approach, including workplace protections, paternity entitlements, and community-based norm change programmes, is essential to redistributing caregiving responsibilities more equitably (36, 37).

### 4.3 Economic precarity and child well-being

Economic vulnerability among Adi-Dravidar (Scheduled Caste) mothers in this study cannot be separated from caste-based occupational segregation that historically confined these communities to low-wage, low-status labour. Unlike non-caste-based poverty, this economic exclusion is structurally reproduced across generations through the denial of educational and employment opportunities rooted in caste identity (7). The single mother with higher educational attainment in this study powerfully demonstrates that when caste-based educational exclusion is overcome, caregiving capacity and children's developmental outcomes improve significantly, underscoring that poverty alone is insufficient to explain the disadvantages experienced by these families. These findings align with extensive literature documenting how poverty restricts children's developmental opportunities and reinforces intergenerational disadvantage (38, 39). Economic precarity limits not only material resources but also parental capacity for sustained engagement, creating cascading effects on child outcomes.

The intergenerational dimension of economic precarity deserves particular emphasis. Children growing up in households where mothers are trapped in exhausting, low-wage, caste-segregated labour are not merely experiencing current deprivation, they are being socialized into the same constrained opportunity structures that their mothers inhabit. The absence of economic security limits children's access to educational materials, tutoring, nutritious food, and safe spaces for play and learning, all of which are critical inputs for healthy middle childhood development (38, 39). Furthermore, chronic household financial stress generates a form of ambient adversity that shapes children's neurobiological stress-response systems, with long-term implications for mental health, cognitive functioning, and socio-emotional development (39). Addressing this requires not only immediate income-support measures but also long-term structural reforms targeting caste-based occupational segregation, including reservation enforcement in skilled employment, legal protections against caste discrimination in the labour market, and community-level economic development programmes specifically designed for Scheduled Caste communities.

### 4.4 Educational implications

Educational exclusion among Adi-Dravidar (Scheduled Caste) mothers in this study reflects a distinctly caste-based pattern of marginalization, these mothers were not simply too poor to access education but were historically denied educational opportunities due to intersecting caste and gender discrimination that positioned Dalit girls and women as undeserving of formal education (7, 14). This caste-specific educational exclusion has direct intergenerational consequences, as mothers unable to read or write cannot support their children's academic development regardless of their motivation or commitment. Education represents a critical mechanism for advancing Sustainable Development Goals, particularly poverty reduction (Goal 1), quality education (Goal 4), and gender equality (Goal 5) (40, 41). Educated parents are better positioned to support children's learning and advocate for educational opportunities. However, in contexts where parents themselves were denied education, this intergenerational transmission of disadvantage becomes entrenched. Research demonstrates that maternal education strongly predicts child educational outcomes, health, and cognitive development (42, 43). Interventions must therefore address both current child education and adult literacy to disrupt cycles of educational disadvantage.

The findings further highlight a critical mismatch between school-based educational expectations and the capacities of Adi-Dravidar (Scheduled Caste) mothers. Contemporary schooling systems in India increasingly rely on active parental involvement, homework assistance, communication with teachers, attendance at parent-teacher meetings, as essential components of children's academic support. These expectations implicitly presuppose a level of literacy and institutional confidence that was systematically denied to Dalit mothers through decades of caste and gender-based educational exclusion. This mismatch does not reflect parental disengagement or indifference but rather a structural misalignment between institutional expectations and historically produced parental capabilities (14, 42). Schools operating in communities like Puliyanthope must therefore adapt their engagement models to be inclusive of low-literacy parents, employing oral communication, visual aids, and community mediators to bridge this gap. Concurrently, government-funded adult literacy programmes that are trauma-informed and caste-sensitive are urgently needed to equip mothers with the skills to participate more actively in their children's educational lives.

### 4.5 Physical punishment as a normalized parenting practice in unsafe living environments

The normalization of physical punishment in Adi-Dravidar (Scheduled Caste) households in this study must be understood within the context of caste-based spatial segregation that has concentrated marginalized communities in unsafe, institutionally neglected neighbourhoods, a condition that is not simply a consequence of poverty but of deliberate historical exclusion from safer, better-resourced urban spaces (19). While physical punishment is harmful to child development and well-being (44, 45), in this context it stemmed from pervasive fear regarding neighbourhood safety rather than parental ignorance or neglect. Living in unsafe environments with frequent reports of child abuse and sexual violence against children created constant anxiety among parents, consistent with documented patterns of violence in similar marginalized urban informal settlement contexts (46, 47). Physical discipline became perceived as a protective mechanism ensuring obedience and keeping children confined to home. This finding underscores how environmental conditions shape parenting practices, with structural violence manifesting in parent-child relationships. Addressing harmful disciplinary practices requires interventions at multiple levels: educating parents about positive discipline techniques, improving neighbourhood safety, and addressing root causes of violence in marginalized communities. Approaches that pathologize parents without addressing structural determinants will prove ineffective.

This finding also reveals an important paradox of structural violence: the very conditions imposed upon Adi-Dravidar (Scheduled Caste) families by caste-based spatial segregation generate parenting behaviours that further expose children to developmental harm. Mothers who resort to physical discipline do so not from a deficit of care but from an excess of fear, fear that is a rational response to genuinely dangerous living environments created by institutional neglect and caste-based relegation to unsafe urban peripheries. This framing is critical for intervention design. Parenting programmes that frame physical punishment purely as a behavioural or attitudinal problem without acknowledging its structural roots risk pathologizing and alienating the very parents they seek to support (44, 45). Effective approaches must combine positive parenting education with concrete environmental safety improvements, better street lighting, police accountability, survivor-centred responses to gender-based violence, and investment in community infrastructure, so that mothers have realistic alternatives to physical confinement as a child-protection strategy. Only by addressing both the proximate behaviour and its structural determinants can such programmes achieve sustainable impact.

### 4.6 Social isolation and absent support systems

Social isolation among Adi-Dravidar (Scheduled Caste) mothers in this study extends beyond the fragmentation of family networks common in urban migration contexts. Caste-based stigmatization of Puliyanthope as a criminalized neighbourhood has systematically eroded residents' access to broader social networks, institutional support, and civic participation, creating a form of caste-specific social isolation that compounds the caregiving burden in ways distinct from poverty-based isolation alone (7, 19). Social support is critical for maternal mental health, parenting capacity, and child outcomes (48, 49). Mothers experiencing social isolation face elevated risks of depression, anxiety, and burnout, which directly impact children's emotional well-being and development. The absence of affordable, accessible childcare services represents a critical policy gap requiring urgent attention.

The erosion of social support networks in contexts like Puliyanthope has compounding effects that extend well beyond individual maternal mental health. Social networks function as informal safety nets, providing emergency childcare, emotional support during crises, shared knowledge about child health and development, and collective advocacy for community resources. When caste-based stigmatization systematically excludes Adi-Dravidar (Scheduled Caste) mothers from broader civic and social participation, these protective functions are lost, leaving families more vulnerable to every other form of adversity they face (48, 49). Rebuilding these networks requires both community-level and institutional interventions. Community centres, women's self-help groups, and caste-sensitive social work services can provide structured spaces for rebuilding social connection and mutual support. Maternal mental health services, currently almost entirely absent in informal settlement contexts, must also be integrated into primary health care provision as a matter of priority.

### 4.7 Maternal resilience

The remarkable resilience demonstrated by Adi-Dravidar (Scheduled Caste) mothers in this study must be understood not as a cultural characteristic of marginalized communities but as a response to structural conditions imposed by intersecting caste, gender, and economic inequalities. Framing resilience as inherent to these mothers' risks obscuring the urgent need for systemic interventions and inadvertently justifying continued institutional neglect of marginalized urban communities. However, these findings caution against romanticizing individual resilience as a substitute for systemic support. Research consistently demonstrates that maternal resilience operating within severe structural constraints is inherently limited in its capacity to protect children's developmental outcomes (31, 32). When poverty, caste-based marginalization, absent paternal involvement, and institutional neglect converge, individual coping strategies prove insufficient. These findings therefore underscore that maternal resilience must be recognized, supported, and structurally enabled rather than assumed or expected.

Recognizing maternal resilience as structurally produced rather than individually intrinsic also reframes the appropriate locus of intervention. When resilience is understood as a personal trait, the logical response is to build individual coping skills through psychological training or motivational programmes. When it is understood as a structural achievement, maintained against extraordinary odds by mothers navigating multiple intersecting disadvantages simultaneously, the appropriate response is to remove structural barriers and provide institutional support (31, 32). This distinction has direct programme design implications. Strengths-based approaches that acknowledge and build upon the adaptive strategies these mothers have already developed, while simultaneously working to reduce the structural burdens that make such strategies necessary, are most likely to be both effective and respectful of participants' dignity. Critically, mothers themselves should be positioned as co-designers and community advocates in any intervention, transforming their structural knowledge of caregiving under adversity into a collective resource for community change.

### 4.8 Policy and practice implications

Findings underscore the need for comprehensive, multi-level interventions addressing maternal and child well-being in marginalized urban informal settlement contexts. These include: (1) Community-Based Childcare Services, establishing accessible, affordable childcare in urban informal settlement communities to reduce maternal burden and enable safer child supervision; (2) Promoting Shared Parenting, gender-transformative interventions engaging fathers through community education programs and paternity policy initiatives (36, 37); (3) Economic Empowerment Programs, microfinance, skills training, and employment support to strengthen household economic security and maternal agency; (4) Adult Education and Literacy, programmes addressing low maternal education levels to enhance parenting capacity and support children's educational engagement; (5) Neighbourhood Safety and Infrastructure, addressing unsafe living conditions, improving sanitation, lighting, and security; and (6) Integrated Social Services, comprehensive support systems linking healthcare, education, social welfare, and mental health services.

Crucially, the effective implementation of these policy recommendations requires moving beyond siloed programmatic responses toward an integrated, community-anchored service delivery model. Current social protection systems in urban India tend to operate in sectoral silos, health departments address maternal health separately from education departments managing school outcomes, while labour departments handle economic programmes with minimal coordination with social welfare services. For families facing intersecting, mutually reinforcing disadvantages, this fragmented approach generates both service gaps and duplication, while placing the burden of navigating multiple bureaucratic systems on mothers who are already overextended (38, 40). An integrated urban family support framework that co-locates childcare, adult education, economic empowerment, mental health support, and community safety programming within accessible community hubs would more effectively address the compounded nature of these families' needs. Furthermore, all such interventions must be explicitly caste-sensitive, involving Dalit community organizations in design and governance to ensure cultural relevance, trust, and accountability to the communities they serve.

### 4.9 Study limitations

This study has several limitations. The qualitative design with 20 participants limits generalizability to other contexts. Findings reflect experiences of Adi-Dravidar (Scheduled Caste) mothers in one Chennai urban informal settlement and may not represent all marginalized communities. Cross-sectional design precludes assessment of long-term child development outcomes. Future research employing mixed methods, larger samples, and longitudinal designs would strengthen evidence base. Additionally, the study focused primarily on mothers' perspectives. Future research should include children's voices and fathers' perspectives to provide more comprehensive understanding of family dynamics and parenting practices in marginalized contexts.

Age data were not obtained from 60% of participants, reflecting common challenges in marginalized communities where formal age documentation may be absent and age disclosure may be affected by limited formal education. This limitation did not affect core study findings related to caregiving practices and maternal experiences.

## 5 Conclusion

This study reveals how maternal caregiving and child well-being in marginalized urban contexts are profoundly shaped by intersecting structural inequalities rooted in caste, gender, and poverty. Adi- Dravidar (Scheduled Caste) mothers in Puliyanthope urban informal settlement face overwhelming challenges managing childcare responsibilities while engaging in precarious informal labour, with minimal support from partners, family, or institutions. Findings demonstrate that child developmental outcomes are directly compromised by maternal time poverty, economic insecurity, limited parental education, unsafe neighbourhoods, and absent support systems. Physical punishment, educational disadvantage, and restricted developmental opportunities reflect how structural violence manifests in children's lives. Critically, the study underscores that inadequate parenting practices in marginalized communities should not be interpreted as individual failings but as outcomes of systemic exclusion and institutional neglect. Maternal resilience, while remarkable, cannot substitute for comprehensive social support and structural reform. Addressing maternal and child well-being in urban informal settlements requires multi-level interventions: establishing community-based childcare services, promoting shared parenting responsibilities, implementing economic empowerment programs, expanding adult education, improving neighbourhood safety, and developing integrated social services. Only through comprehensive approaches addressing root causes of inequality can cycles of disadvantage be disrupted and children's developmental potential realized. Future research should employ longitudinal designs examining child developmental trajectories, include children's and fathers' perspectives, and evaluate intervention effectiveness in marginalized urban contexts. Additionally, comparative studies across different urban informal settlement communities and caste groups would strengthen understanding of context-specific needs and effective intervention strategies.

## Data Availability

The raw data supporting the conclusions of this article will be made available by the authors, without undue reservation.
